# Supplementary material for: Quantum phase classification via partial tomography-based quantum hypothesis testing
Source: Sci Rep. 2026 Feb 2;16:4555. doi: 10.1038/s41598-025-34610-2 (PMC12868643; doi:10.1038/s41598-025-34610-2)
Supplement: Supplementary file 1 — Supplementary Information. [file 41598_2025_34610_MOESM1_ESM.pdf]

Supplementary Information: Quantum phase classification via partial tomography-based quantum hypothesis testing

Akira Tanji[1,*], Hiroshi Yano[2], and Naoki Yamamoto[1,2,+]

[1]Department of Applied Physics and Physico-Informatics, Keio University, Hiyoshi 3-14-1, Kohoku, Yokohama 223-8522, Japan
[2]Keio Quantum Computing Center, Keio University, Hiyoshi 3-14-1, Kohoku, Yokohama 223-8522, Japan
[*]tanjikeio@keio.jp
[+]yamamoto@appi.keio.ac.jp

A Settings of numerical simulations

We first describe how phase classification was performed using the methods employed in the numerical results presented in the main text.

Order parameter. The classification using the order parameter, detailed in Section D.2, involves applying projective measurements defined by the observables O_{FM} for the Trivial vs. FM case and O_{SPT} for the Trivial vs. SPT case in the main text. These measurements are performed on the test data, and are utilized for testing whether the expectation values of the order parameter are zero or not, using the Bayesian test for the Trivial vs. FM case and the classical Neyman-Pearson test for the Trivial vs. SPT case.

Exact QCNN. The Exact QCNNs used in our numerical simulation are shown in Fig. 1. The Exact QCNN for the FM phase was developed in this study, whereas the one for the SPT phase was taken from Ref.[1]. Both were constructed based on theoretical insights, such as order parameters and the renormalization group. For classification using the Exact QCNN, also detailed in Section D.3, the test data are input into the circuit shown in Fig. 1(a) for the Trivial vs. FM case and in Fig. 1(b) for the Trivial vs. SPT case. The output expectation values are then evaluated using the classical Neyman-Pearson test to test whether they exceed 0.5. Test data with output values above 0.5 are classified as label $y^{(i)} = 1$, and those not exceeding 0.5 as label $y^{(i)} = 0$.

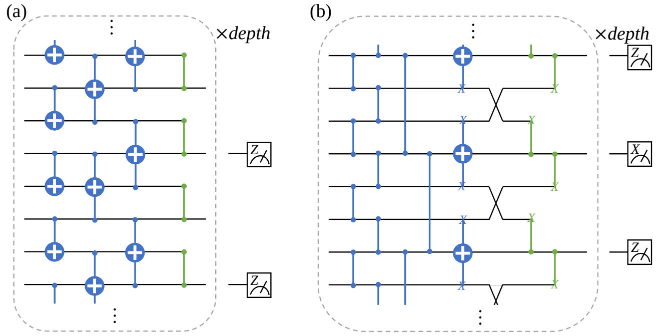

Figure 1. Exact QCNN circuits for (a) the FM and (b) SPT phases. The details of (a) are provided in Section D.3, while (b) is proposed in Ref.[1]. The circuit consists of convolutional layers (blue) and pooling layers (green, reduce the qubits), repeated for a specified depth, followed by Pauli measurements on the remaining qubits.

QCNN. The QCNN is trained using the ansatz shown in Fig. 2, initialized randomly. The training data are input, and the circuit parameters θ are updated over epochs using the SPSA optimizer to minimize the MSE loss

$$\mathrm{MSE}(\theta) = \frac{1}{N_{\mathrm{train}}} \sum_{i=1}^{N_{\mathrm{train}}} \left(f(\theta, \rho^{(i)}) - y^{(i)} \right)^2, \tag{1}$$

where $f(\theta,\rho^{(i)})$ denotes the output expectation value when the training data $\rho^{(i)}$ is input into the QCNN parameterized by θ. This training is performed for both the Trivial vs. FM and the Trivial vs. SPT cases. The trained QCNN classifies the test data using the classical Neyman-Pearson test, following the same procedure as described for the Exact QCNN: output values above 0.5 are assigned label $y^{(i)}=1$, and those not exceeding 0.5 are assigned label $y^{(i)}=0$.

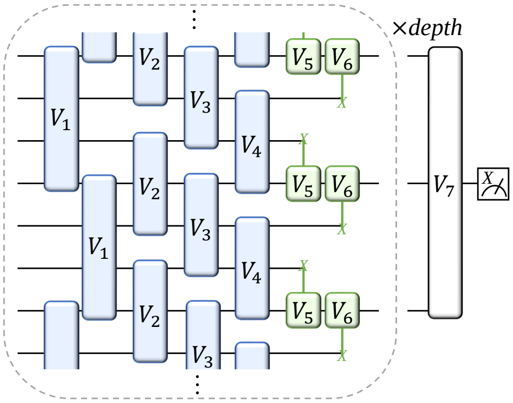

Figure 2. QCNN ansatz proposed in Ref.[1]. The circuit consists of convolutional layers (blue) and pooling layers (green, reduce the qubits), repeated for a specified depth, followed by fully connected layer (black) and Pauli measurements on the remaining qubits. The unitaries are parameterized as $V=\exp(-i\sum_j \theta_j\Lambda_j)$, where $\{\Lambda_j\}$ are generalized Gell-Mann matrices and $\{\theta_j\}$ are real parameters.

low-weight QCNN. The low-weight QCNN uses classical shadow snapshots of the training data obtained through quantum experiments and is trained by classical simulation using only the low-weight components of the Heisenberg-evolved observables, i.e., Pauli basis elements with a small number of non-identity qubits. Since the snapshots are collected once and can be reused for any number of epochs, the low-weight QCNN is trained for 300 epochs, where the validation loss reaches a stable level. The low-weight QCNN limit the weight of Pauli basis elements up to three, employs random Pauli measurements for classical shadow, and the other settings such as the ansatz and loss function follow the same as the QCNN.

B Majority vote

After performing the partitioned quantum Neyman–Pearson tests on each subsystem, the individual subsystem predictions must be combined by a classical post-processing step. In our numerical simulations we use a simple majority vote. In this section we justify this choice by showing that, under an exponential clustering assumption, majority vote reduces the prediction variance. We also discuss alternative classical aggregation methods.

The following assumption is known to hold, e.g., for unique ground states of local and gapped Hamiltonians[2,3]. It is a typical property in simple models but may fail at critical points where phase transitions occur. Consequently, while it supports our design choice, it does not fully cover all regimes probed in our simulations.

Assumption 1 (Exponential clustering[2,3]). *Let ρ be the density operator of the entire system. For disjoint subsystems $\mathscr{H}_X, \mathscr{H}_Y$ supported on site-sets X,Y, define the reduced density matrices by $\rho_X = Tr_{\neg X}(\rho)$, $\rho_Y = Tr_{\neg Y}(\rho)$, and $\rho_{XY} = Tr_{\neg(X\cup Y)}(\rho)$, where $\neg X$ denotes the complement of X in the entire system. Let $r_{XY} = \min_{x\in X, y\in Y} \mathrm{dist}(x,y)$ be the graph-theoretic distance between X and Y, and let $\xi > 0$ denote the correlation length. For any local observables A_X on $\mathscr{H}_X$ and B_Y on $\mathscr{H}_Y$ with $\|A_X\| \le 1$ and $\|B_Y\| \le 1$, correlations decay exponentially with distance,*

$$\left|\mathrm{Tr}\left[(A_X \otimes B_Y)\,(\rho_{XY} - \rho_X \otimes \rho_Y)\right]\right| \le \mathrm{Const.} \times e^{-r_{XY}/\xi}\,. \tag{2}$$

Treating each partitioned quantum Neyman–Pearson test as a weak classifier, the overall procedure can be viewed as a form of model ensemble learning. In classical machine learning, a simple majority vote is well known to be a natural ensemble method for classification problems, and its effectiveness is governed by the correlations among weak classifiers. Under Asm. 1, the covariance between the i- and j-th subsystems decays exponentially with their separation r_{ij} and the inverse correlation length $1/\xi$. As a result, when aggregating their predictions by majority vote, the estimator exhibits variance reduction analogous to model ensembling methods. In particular, when ξ is sufficiently small, the variance scales as $\mathscr{O}(1/M)$ with $M = L/k$ the number of subsystems.

Theorem 1 (Variance reduction by the majority vote on a d-dimensional model). *Let the $M = L/k$ subsystems be arranged on a d-dimensional lattice, and let $\{S_j^{(0)}, S_j^{(1)}\}$ be a two-outcome POVM on subsystem $j \in \{1, \ldots, M\}$. For each j, define a random variable $Y_j \in \{0,1\}$ whose marginal distribution is Bernoulli with $\Pr(Y_j = 1) = \mathrm{Tr}(S_j^{(1)} \rho_j)$, where ρ_j is the RDM on subsystem j. Let the average estimator be $\bar{Y} = \frac{1}{M}\sum_{j=1}^{M} Y_j$, which serves as the majority vote estimator when thresholded at 0.5. Under Asm. 1,*

$$\mathrm{Var}(\bar{Y}) \;\le\; \frac{\sigma^2}{M}\left[1 + \frac{C}{\sigma^2}\left\{\left(\frac{1+e^{-1/\xi}}{1-e^{-1/\xi}}\right)^d - 1\right\}\right], \tag{3}$$

where $\sigma^2 = \max_j \mathrm{Var}(Y_j)$ and C is the maximum constant appearing in Asm. 1 taken over all subsystems. In particular, for small correlation length ξ, the dominant scaling is $\mathrm{Var}(\bar{Y}) \lessapprox \sigma^2/M$.

Proof. Write $\sigma_j^2 = \mathrm{Var}(Y_j)$ and $\mathrm{cov}_{i,j} = \mathrm{Cov}(Y_i, Y_j)$. By Asm. 1 and $\|S_j^{(1)}\| \le 1$,

$$|\mathrm{cov}_{i,j}| = \big|\mathbb{E}[Y_i Y_j] - \mathbb{E}[Y_i]\mathbb{E}[Y_j]\big| = \big|\mathrm{Tr}\big[(S_i^{(1)} \otimes S_j^{(1)})(\rho_{ij} - \rho_i \otimes \rho_j)\big]\big| \le \mathrm{Const.} \times e^{-r_{ij}/\xi}, \tag{4}$$

and thus

$$\mathrm{Var}(\bar{Y}) = \frac{1}{M^2}\Big(\sum_{j=1}^{M} \sigma_j^2 + 2\sum_{1\le i<j\le M} \mathrm{cov}_{i,j}\Big) \;\le\; \frac{\sigma^2}{M} + \frac{2C}{M^2}\sum_{1\le i<j\le M} e^{-r_{ij}/\xi}. \tag{5}$$

For the d-dimensional lattice with L_1 distance, the generating function identity

$$\sum_{r=1}^{\infty} N_d(r)\, q^r = \left(\frac{1+q}{1-q}\right)^d - 1, \tag{6}$$

holds for any scalar q with $|q| < 1$, where $N_d(r)$ denotes the number of sites at distance r from a given site (see, e.g., Thm. 2.7 of [4], from which it follows by a straightforward calculation). Using this,

$$\frac{1}{M^2}\sum_{1\le i<j\le M} e^{-r_{ij}/\xi} \;\le\; \frac{1}{M^2}\sum_{r=1}^{\infty} e^{-r/\xi}\,\frac{1}{2}\sum_{i=1}^{M} N_d(r) = \frac{1}{2M}\sum_{r=1}^{\infty} N_d(r) e^{-r/\xi}, \tag{7}$$

where the factor $1/2$ accounts for the fact that each unordered pair (i,j) is counted twice when summing over all i. Substituting this bound completes the proof of Eq. (3). □

Although majority vote is not necessarily optimal in all settings, under the exponential clustering assumption it behaves as a standard and justified ensemble method. Finally, we note that the majority vote is obtained simply by thresholding the average estimator $\bar{Y}$ at 0.5. Thus, the variance reduction of $\bar{Y}$ directly justifies the stability of the majority vote. Moreover, in the extreme uncorrelated case (i.e., $\forall i, j,\ \mathrm{cov}_{i,j} = 0$) for odd M, the majority vote estimator $X \sim \mathrm{Bernoulli}(\Pr\{\bar{Y} > 0.5\})$ satisfies $\big|\mathbb{E}[X] - 0.5\big| \;\ge\; \big|\mathbb{E}[\bar{Y}] - 0.5\big|$, which follows from the ultra log-concavity of Poisson binomial distributions[5,6]. This provides a futher rationale for adopting the majority rule. We note that the above result concerns the variance of the estimator given fixed system and subsystem sizes L and k. If one instead increases M by decreasing k, each subsystem maintains less information about the entire state, which may affect classification accuracy. This trade-off is separate from the variance reduction analyzed in the theorem.

Beyond the uniform majority vote, we may also consider a weighted variant

$$\bar{Y}_w = \frac{1}{\|w\|_1}\sum_{j=1}^{M} w_j Y_j. \tag{8}$$

Such weights may be chosen, for example, to reduce finite-size effects by assigning smaller weights to subsystems near the boundary and larger weights to those in the bulk. Alternatively, weights may be determined from subsystem performance estimates obtained from RDMs estimated on training data, which does not require additional copies of the quantum states. When sufficient test copies are available, the weights may be adjusted based on confidence estimates obtained at inference time.

C Additional numerical simulations

Here, we present additional results to complement the numerical findings presented in the main text.

C.1 Robustness under depolarizing noise

To evaluate the robustness of our method under a standard noise model, we consider global depolarizing noise defined by

$$D_p(\rho) = (1-p)\,\rho + p\,\frac{I}{d}, \tag{9}$$

where ρ is an arbitrary quantum state, p is the depolarizing probability, d is the dimension of ρ, and I is the identity operator. In the training step, depolarizing noise with total probability p is applied both to the training states $\{\rho^{(i)}\}_{i=1}^{N_{\text{train}}}$ and to the tomography unitary $\bigotimes_{j=1}^{L/k} U_j$. That is, we perform measurements in the computational basis on the noisy states $D_p\Big((\bigotimes_{j=1}^{L/k} U_j)\,\rho^{(i)}\,(\bigotimes_{j=1}^{L/k} U_j)^\dagger \Big)$ to obtain snapshots for the partial state tomography. In the test step, depolarizing noise with total probability p is applied both to the test states $\{\rho_{\text{test}}^{(i)}\}_{i=1}^{N_{\text{test}}}$ and to the unitary $\bigotimes_{j=1}^{L/k} [\,|\lambda_{j,1}\rangle, \ldots, |\lambda_{j,2^k}\rangle]^\dagger$ used in the partitioned quantum Neyman-Pearson test.

The error probabilities when our method is run on a 27-qubit system ($L = 27$) under the depolarizing noise are shown in Fig. 3. From the panel (a), where only training noise is applied, we observe that the performance of our method deteriorates very little. A rough analysis suggests that this robustness arises because, when depolarizing noise acts on both ρ and σ in the POVM element of the quantum Neyman-Pearson test defined in the main text (with $a = 0$ and $n = 1$), we obtain

$$\{D_p(\rho) - D_p(\sigma) > 0\} = \{((1-p)\rho + p\frac{I}{d}) - ((1-p)\sigma + p\frac{I}{d}) > 0\} = \{(1-p)(\rho - \sigma) > 0\} = \{\rho - \sigma > 0\}, \tag{10}$$

indicating that the POVM remains unchanged. Although the eigenvalues of $\rho - \sigma$ are scaled by a factor of $1 - p$, the sign discrimination of these eigenvalues is handled by classical processing in our method and is therefore largely unaffected. On the other hand, some performance degradation is observed in the panel (b), where noise is applied in both the training and test steps, as expected given that the evaluation considers the error probabilities for a single copy of the test data. This can be understood as follows. Let $\alpha_1^{(p)}$ and $\beta_1^{(p)}$ be the Type-I and Type-II error probabilities for a single copy under depolarizing noise with probability p in the test step, and then, using the definitions of the error probabilities as given in the main text, we have

$$\alpha_1^{(p)} = (1-p)\alpha_1^{(p=0)} + p\,\mathrm{Tr}\left(\frac{I}{d}(I - M_1)\right) = (1-p)\alpha_1^{(p=0)} + p\left(1 - \mathrm{Tr}\left(\frac{I}{d}M_1\right)\right), \tag{11}$$

$$\beta_1^{(p)} = (1-p)\beta_1^{(p=0)} + p\,\mathrm{Tr}\left(\frac{I}{d}M_1\right), \tag{12}$$

for any classification method M_1, not just our method. Thus, the results in the panel (b) follow naturally from the definition of the error probabilities and the presence of depolarizing noise. Such degradation could potentially be mitigated by improving the state preparation of test data or by employing error mitigation techniques.

C.2 Error probabilities for $n_{\text{ent}} > 1$

The approximate quantum Neyman-Pearson test $\{S_j^{(0)}(n_{\text{ent}}, a), S_j^{(1)}(n_{\text{ent}}, a)\}$ in the main text involves an entangled measurement on n_{ent} copies of quantum states. However, all numerical simulations in the main text were performed with $n_{\text{ent}} = 1$, as our method requires the eigendecomposition of kn_{ent} qubits, which becomes exponentially difficult with increasing n_{ent}. This section demonstrates that increasing n_{ent} beyond 1 provides limited benefits.

For $n_{\text{ent}} > 1$, the training steps 1 to 3 remain unchanged. In training step 4, the eigenvalues and eigenvectors of $\hat{\rho}_j^{\otimes n_{\text{ent}}} - e^{n_{\text{ent}} a}\hat{\sigma}_j^{\otimes n_{\text{ent}}}$ are calculated instead of $\hat{\rho}_j - e^a \hat{\sigma}_j$. In test step 2, measurements using the POVMs corresponding to the quantum Neyman-Pearson test are performed on n_{ent} copies of the test data.

For $n_{\text{ent}} = 1$ and 3, the Type-I and Type-II error probabilities for $n = 3$ copies of test data, α_3 and β_3, are shown in Fig. 4. In the partial tomography of training data, if an infinite number of copies are used, the resulting states are obtained by performing partial traces that retain only each k-qubit group. The infinite-shots results therefore correspond to the quantum Neyman-Pearson test constructed using these partial traced states for each k-qubit group. Fig. 4 indicates that there is no significant difference in error probabilities between $n_{\text{ent}} = 1$ and 3, regardless of finite or infinite shots and whether the system size is $L = 15$ or 27 qubits. However, for $n_{\text{ent}} = 3$, eigendecomposition and gate construction for the quantum Neyman-Pearson test are required for $kn_{\text{ent}} = 2 \times 3 = 6$ qubits, resulting in a significant increase in classical computational complexity. While larger n_{ent} might reduce error probabilities, the exponential increase in classical computational time with n_{ent} makes $n_{\text{ent}} = 1$ the most practical choice.

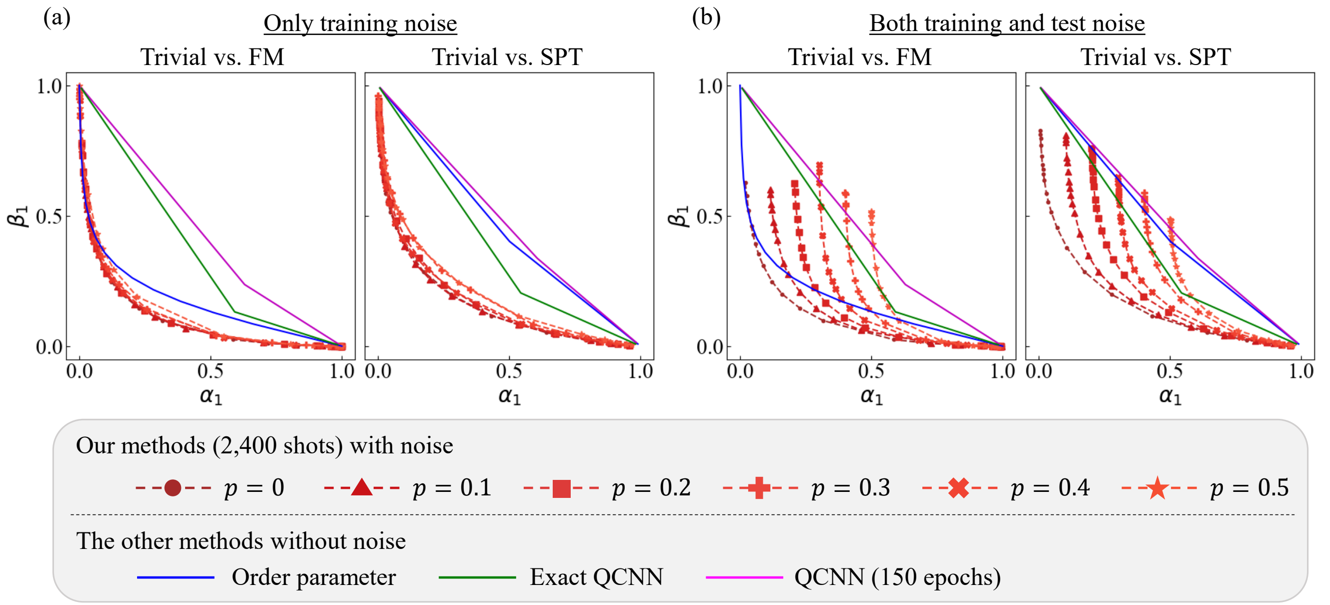

Figure 3. Type-I and Type-II error probabilities, α_1 and β_1, under global depolarizing noise with probability p for single-copy test data ($n = 1$) on $L = 27$ qubits in the Trivial vs. FM and Trivial vs. SPT cases. The results for the other methods are identical to those in the main text, with no noise applied. The panel (a) shows the results with noise applied only in the training step, and the panel (b) shows the results with noise applied in both the training and test steps.

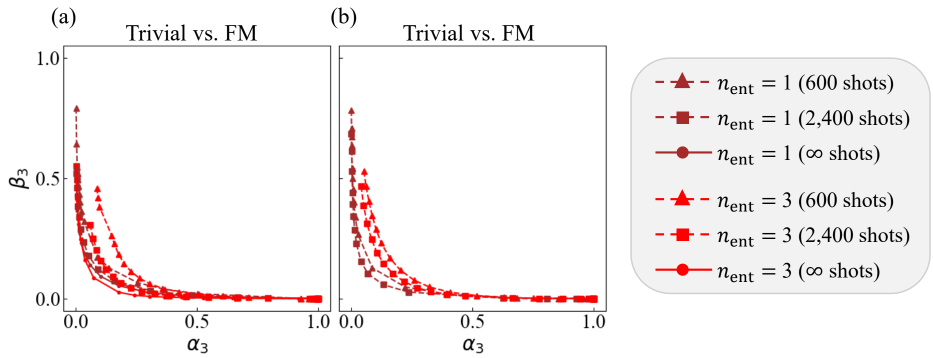

Figure 4. Type-I and Type-II error probabilities, α_3 and β_3, for $n = 3$ copies of test data with $n_{\mathrm{ent}} = 1$ and 3. Panel (a) results for an $L = 15$ qubit system, and panel (b) results for an $L = 27$ qubit system, both in the Trivial vs. FM case ($k = 2$). The total number of training data copies is 600 or 2,400, with infinite-copy results also included for (a).

C.3 Two-dimensional model

In this section, we demonstrate that our method could work effectively for two-dimensional models where the dividing strategy is non-trivial, even with a simple dividing method. We conduct numerical simulations for quantum phase classification of the two-dimensional Toric code Hamiltonian with magnetic fields

$$H = H_{\mathrm{TC}} - h_X \sum_{i=1}^{L} X_i - h_Z \sum_{i=1}^{L} Z_i, \tag{13}$$

where X_i (Z_i) are the Pauli X (Z) operators on the i-th qubit, and h_X (h_Z) are the tunable strengths of the magnetic fields in the X (Z) direction. Here, the Toric code Hamiltonian is

$$H_{\mathrm{TC}} = -\sum_p A_p - \sum_s B_s, \tag{14}$$

where $A_p = \prod_{i \in \mathrm{plaquette}(p)} X_i$ and $B_s = \prod_{i \in \mathrm{star}(s)} Z_i$ are the plaquette and star operators, respectively. The ground states of this Hamiltonian undergo a quantum phase transition between the topological phase of the Toric code and the trivial magnetic phase[7,8]. For instance, changing h_Z at $h_X = 0$ induces a phase transition at $h_Z = 0.34$, and at $h_X = 0.1$, the phase transition occurs at $h_Z = 0.35$[7]. We classify the quantum phases using the Exact QCNN and our method. The test data, which are quantum states we want to phase classify, are common between the two methods and consist of 100 ground states near $h_X = 0.1$ and $h_Z = 0.35$. The training data required for our method are ground states along $h_X = 0$ with h_Z evenly divided into 20 points in $[0, 0.68]$, with labels assigned as $y^{(i)} = 0$ for the trivial magnetic phase and $y^{(i)} = 1$ for the topological phase. For numerical simulations, we use the MPS mapped to one dimension, as illustrated by the red dash-dot line in Fig. 5(a). Approximate ground states are prepared using the finite-size DMRG algorithm with a maximum bond dimension of 1500.

We describe the settings for each method.

Exact QCNN. For classification using the Exact QCNN, detailed in Section D.3, the test data are input into the circuit proposed in Ref.[7], and then the output expectation values are tested whether they exceed 0.5 using the classical Neyman-Pearson test. Test data with output values above 0.5 are classified as label $y^{(i)} = 1$, and those not exceeding 0.5 as label $y^{(i)} = 0$. The limited system size in our numerical simulation ($L = 18$ qubits) restricts the circuit depth, which may hinder the Exact QCNN from achieving its expected performance.

Our method. For our method, we use $k = 2$ qubit groups and the algorithm described in the Methods section of the main text. We divide the quantum many-body states into groups of $k = 2$ qubits based on the one-dimensional ordering shown in Fig. 5(a), where neighboring qubits are grouped sequentially from one end of the chain. Other settings, such as the partial tomography method, follow the configurations in the main text.

The Type-I and Type-II error probabilities, α_n and β_n, for the test data on $L = 18$ qubits are shown in Figs. 5(b)(c). These figures indicate that our method achieves sufficient performance even with simple dividing strategy. Note that the Exact QCNN is provided with prior knowledge of quantum phases but no training data, whereas our method uses training data without prior knowledge of the quantum phases, and this difference in problem setup precludes a direct comparison of performance.

D Details of methods other than our method

Here, we provide detailed explanations of the methods other than our method used for comparison in the numerical simulations presented in the main text.

D.1 Classical hypothesis testing

Classical hypothesis testing[9–12] serves as a core approach in statistical inference, developed to evaluate competing hypotheses based on sample data. In this framework, two hypotheses are considered: the null hypothesis H_0, which represents a default state or baseline assumption, and the alternative hypothesis H_1, which represents an effect or deviation from the baseline. Classical hypothesis testing procedures are used to make decisions under uncertainty, particularly in fields like scientific research, quality control, and medical diagnostics, where making reliable decisions based on observed data is essential.

The decision-making process involves controlling error probabilities associated with incorrect decisions. Specifically, a Type-I error occurs if we reject H_0 when it is true, with probability denoted by α, and a Type-II error occurs if we fail to reject H_0 when H_1 is true, with probability denoted by β. Balancing these errors is central to classical testing; the significance level α is usually predefined, while the power of the test, $1 - \beta$, represents the probability of correctly rejecting H_0 when H_1 is true.

A fundamental result in hypothesis testing is the Neyman-Pearson lemma, which provides a criterion for constructing the most powerful test for simple hypotheses at a given significance level α.

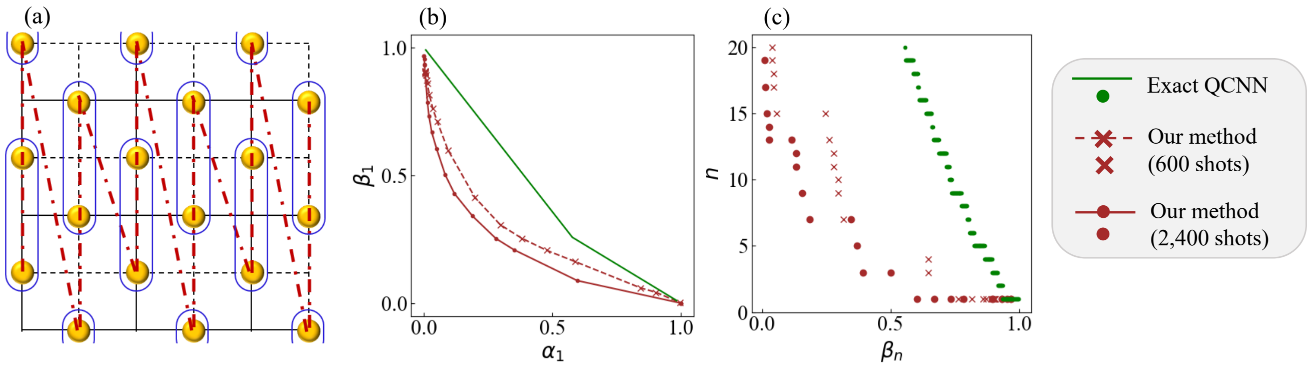

Figure 5. (a) Toric code lattice with $L = 18$ qubits. The red dashed-dotted line indicates the mapping order to one dimension for the MPS representation, and the blue circles represent the dividing strategy used in our method. (b)(c) Type-I and Type-II error probabilities, α_n and β_n, for the test data in the Exact QCNN and our method on $L = 18$ qubits. Panel (b) shows the error probabilities α_1 and β_1 for a single-copy test dataset ($n = 1$), whereas panel (c) shows the number of test data copies n required to achieve β_n under the condition $\alpha_n \leq 5\,\%$. The total number of training data copies used for our method is 600 or 2,400, while the Exact QCNN is the circuit proposed in Ref.[7], which does not require training. It is possible that the Exact QCNN does not perform sufficiently due to the structural constraints on the $L = 18$ qubit system size.

Lemma 1 (Neyman-Pearson Lemma[10]). *Let θ be a parameter determining the probability distribution of a random variable X, which has a probability density $p(x \mid \theta)$. Consider the simple null hypothesis $H_0 : \theta = \theta_0$ and the simple alternative hypothesis $H_1 : \theta = \theta_1$. Define the likelihood ratio as*

$$\Lambda(x) = \frac{p(x \mid \theta_1)}{p(x \mid \theta_0)}. \tag{15}$$

A test ϕ of the form

$$\phi(x) = \begin{cases} 1, & \text{if } \Lambda(x) > c \\ \gamma, & \text{if } \Lambda(x) = c \\ 0, & \text{if } \Lambda(x) < c \end{cases}, \tag{16}$$

exists such that it is the most powerful test at level α, maximizing the power $1 - \beta$. Here, c is the smallest constant satisfying $\Pr\{\Lambda(X) > c \mid \theta = \theta_0\} \leq \alpha$, *and* $\gamma \in [0,1]$ *is chosen to satisfy* $\Pr\{\Lambda(X) > c \mid \theta = \theta_0\} + \gamma \Pr\{\Lambda(X) = c \mid \theta = \theta_0\} = \alpha$.

On the other hand, it is challenging to find a uniformly most powerful (UMP) test for all parameter values under composite hypotheses. However, under specific conditions such as a monotone likelihood ratio (MLR), it is possible to construct the UMP test. Here, the MLR is defined as a likelihood ratio such that the probability distribution differs for any $\theta_0 < \theta_1$ and the ratio $p(x|\theta_1)/p(x|\theta_0)$ is a non-decreasing function of a real-valued function $T(x)$. The Neyman-Pearson lemma 1 is extended to composite hypotheses with MLR as the following lemma.

Lemma 2 ([11]). *Let θ be a real parameter, and let X be a random variable with a probability density $p(x \mid \theta)$ with MLR in a statistic $T(x)$. Consider the following hypotheses:*

(i) $H_0 : \theta = \theta_{\text{th}}, \quad H_1 : \theta > \theta_{\text{th}}$
(ii) $H_0 : \theta \leq \theta_{\text{th}}, \quad H_1 : \theta > \theta_{\text{th}}$.

In both cases (i) *and* (ii)*, there exists a UMP test ϕ of the form*

$$\phi(x) = \begin{cases} 1, & \text{if } T(x) > c \\ \gamma, & \text{if } T(x) = c \\ 0, & \text{if } T(x) < c \end{cases}, \tag{17}$$

at level α, maximizing the power $1 - \beta$ for all θ. Here, c is the smallest constant satisfying $\Pr\{T(X) > c \mid \theta = \theta_{\text{th}}\} \leq \alpha$, *and* $\gamma \in [0,1]$ *is chosen to satisfy* $\Pr\{T(X) > c \mid \theta = \theta_{\text{th}}\} + \gamma \Pr\{T(X) = c \mid \theta = \theta_{\text{th}}\} = \alpha$.

D.2 Order parameter

Classical Neyman-Pearson test using the order parameter of the SPT phase In the context of Trivial vs. SPT, the classification between the trivial phase and SPT phase is achieved by testing whether the expectation value of the order parameter O_{SPT} is zero or not. To establish this, we first prove the following theorem (case (i) is applied in this subsection, while case (ii) is used in the next subsection) concerning the task of testing the expectation value of a Pauli string through projective measurements.

Theorem 2. *Let O be a general Pauli string represented by the spectral decomposition*

$$O = (+1)\Pi_+ + (-1)\Pi_-, \tag{18}$$

where $\Pi_\pm$ are projection operators onto the eigenspaces corresponding to eigenvalues ± 1. For a given quantum state ρ, consider the projective measurement described by O, with probabilities $p(\pm 1) = \mathrm{Tr}(\rho \Pi_\pm)$. Suppose this measurement is performed n times, and let x denote the number of times the eigenvalue $+1$ is observed. Define $\langle O \rangle = \mathrm{Tr}(\rho O) = p(+1) - p(-1)$ as the expectation value of the Pauli string O. Consider the following hypotheses:

(i) $H_0 : \langle O \rangle = \langle O \rangle_{\mathrm{th}}, \quad H_1 : \langle O \rangle > \langle O \rangle_{\mathrm{th}}$
(ii) $H_0 : \langle O \rangle \leq \langle O \rangle_{\mathrm{th}}, \quad H_1 : \langle O \rangle > \langle O \rangle_{\mathrm{th}}$.

In both cases (i) *and* (ii)*, there exists a UMP test ϕ of the form*

$$\phi(x) = \begin{cases} 1, & \text{if } x > c \\ \gamma, & \text{if } x = c \\ 0, & \text{if } x < c \end{cases}, \tag{19}$$

at level α, maximizing the power $1-\beta$ for all $\langle O \rangle$. Here, c is the smallest non-negative integer satisfying $\Pr(x > c \mid \langle O \rangle = \langle O \rangle_{\mathrm{th}}) \leq \alpha$, and $\gamma \in [0,1]$ is chosen to satisfy $\Pr(x > c \mid \langle O \rangle = \langle O \rangle_{\mathrm{th}}) + \gamma \Pr(x = c \mid \langle O \rangle = \langle O \rangle_{\mathrm{th}}) = \alpha$.

Proof. The random variable X, which is the number of times that the eigenvalue $+1$ is observed in n projective measurements described by O, follows a binomial distribution

$$p(x \mid n, p_{\mathrm{bin}}) = \binom{n}{x} p_{\mathrm{bin}}^x (1 - p_{\mathrm{bin}})^{n-x}, \tag{20}$$

where $p_{\mathrm{bin}} = p(+1) = \frac{1+\langle O \rangle}{2}$. In case (i), the hypotheses are rewritten as

$$H_0 : p_{\mathrm{bin}} = p_0 = \frac{1 + \langle O \rangle_{\mathrm{th}}}{2}, \quad H_1 : p_{\mathrm{bin}} = p_1 > \frac{1 + \langle O \rangle_{\mathrm{th}}}{2}, \tag{21}$$

and, in case (ii), the hypotheses are

$$H_0 : p_{\mathrm{bin}} = p_0 \leq \frac{1 + \langle O \rangle_{\mathrm{th}}}{2}, \quad H_1 : p_{\mathrm{bin}} = p_1 > \frac{1 + \langle O \rangle_{\mathrm{th}}}{2}. \tag{22}$$

The likelihood ratio for these hypotheses is

$$\Lambda(x) = \frac{p(x \mid p_{\mathrm{bin}} = p_1)}{p(x \mid p_{\mathrm{bin}} = p_0)} = \frac{\binom{n}{x} p_1^x (1-p_1)^{n-x}}{\binom{n}{x} p_0^x (1-p_0)^{n-x}} = \left(\frac{p_1(1-p_0)}{p_0(1-p_1)} \right)^x \left(\frac{1-p_1}{1-p_0} \right)^n. \tag{23}$$

Since $p_1 > p_0$, it follows that

$$\frac{p_1(1-p_0)}{p_0(1-p_1)} > \frac{p_0(1-p_1)}{p_0(1-p_1)} = 1. \tag{24}$$

Thus, $\Lambda(x)$ is a monotonically increasing function of the statistic $T(x) = x$, i.e., the MLR in $T(x) = x$. By Lemma 2, the test function defined in Eq. (19) is the UMP test for all $\langle O \rangle$ in both cases (i) and (ii). At a given significance level α, c is the smallest non-negative integer satisfying

$$\Pr(x > c \mid \langle O \rangle = \langle O \rangle_{\mathrm{th}}) = \Pr\left(x > c \mid p_{\mathrm{bin}} = \frac{1 + \langle O \rangle_{\mathrm{th}}}{2} \right) = \sum_{x>c} \binom{n}{x} \left(\frac{1 + \langle O \rangle_{\mathrm{th}}}{2} \right)^x \left(\frac{1 - \langle O \rangle_{\mathrm{th}}}{2} \right)^{n-x} \leq \alpha, \tag{25}$$

and $\gamma \in [0,1]$ is chosen to satisfy

$$\begin{aligned}
&\Pr(x > c \mid \langle O \rangle = \langle O \rangle_{\text{th}}) + \gamma \Pr(x = c \mid \langle O \rangle = \langle O \rangle_{\text{th}}) \\
=&\Pr\left(x > c \mid p_{\text{bin}} = \frac{1+\langle O \rangle_{\text{th}}}{2}\right) + \gamma \Pr\left(x = c \mid p_{\text{bin}} = \frac{1+\langle O \rangle_{\text{th}}}{2}\right) \\
=&\sum_{x>c} \binom{n}{x} \left(\frac{1+\langle O \rangle_{\text{th}}}{2}\right)^x \left(\frac{1-\langle O \rangle_{\text{th}}}{2}\right)^{n-x} + \gamma \binom{n}{c} \left(\frac{1+\langle O \rangle_{\text{th}}}{2}\right)^c \left(\frac{1-\langle O \rangle_{\text{th}}}{2}\right)^{n-c} \\
=&\alpha.
\end{aligned} \tag{26}$$

□

By performing the projective measurement described by the order parameter O_{SPT} n times, we test whether the expectation value $\langle O_{\text{SPT}} \rangle$ is zero or not. The test data $\{\rho_{\text{test}}^{(i)}\}_{i=1}^{N_{\text{test}}}$ that we used all have expectation values of O_{SPT} greater than or equal to zero. Thus, the hypothesis corresponding to the trivial phase (labeled $y^{(i)} = 0$) is $H_0 : \text{Tr}(\rho_{\text{test}}^{(i)} O_{\text{SPT}}) = 0$, and the hypothesis corresponding to the SPT phase (labeled $y^{(i)} = 1$) is $H_1 : \text{Tr}(\rho_{\text{test}}^{(i)} O_{\text{SPT}}) > 0$. The test described in case (i) of Thm. 2 with $\langle O \rangle_{\text{th}} = 0$ is therefore UMP for these hypotheses. We refer to this test as the classical Neyman-Pearson test for the SPT phase.

Here, the Type-I error probability is given by

$$\alpha_n^{(i)} = \sum_{x>c} \binom{n}{x} \left(\frac{1+\langle O \rangle^{(i)}}{2}\right)^x \left(\frac{1-\langle O \rangle^{(i)}}{2}\right)^{n-x} + \gamma \binom{n}{c} \left(\frac{1+\langle O \rangle^{(i)}}{2}\right)^c \left(\frac{1-\langle O \rangle^{(i)}}{2}\right)^{n-c}, \tag{27}$$

where $\langle O \rangle^{(i)} = \text{Tr}(\rho_{\text{test}}^{(i)} O_{\text{SPT}})$ for test data labeled $y^{(i)} = 0$. The Type-II error probability is given by

$$\beta_n^{(i)} = \sum_{x<c} \binom{n}{x} \left(\frac{1+\langle O \rangle^{(i)}}{2}\right)^x \left(\frac{1-\langle O \rangle^{(i)}}{2}\right)^{n-x} + (1-\gamma) \binom{n}{c} \left(\frac{1+\langle O \rangle^{(i)}}{2}\right)^c \left(\frac{1-\langle O \rangle^{(i)}}{2}\right)^{n-c}, \tag{28}$$

where $\langle O \rangle^{(i)} = \text{Tr}(\rho_{\text{test}}^{(i)} O_{\text{SPT}})$ for test data labeled $y^{(i)} = 1$. The error probabilities α_n and β_n computed in the main text are the averages of $\alpha_n^{(i)}$ and $\beta_n^{(i)}$ over the test data for each phase, respectively.

Bayesian test using the order parameter of the FM phase In the context of Trivial vs. FM, the classification between the trivial phase and the FM phase is performed by testing whether the expectation value of the order parameter O_{FM}, defined as a linear combination of local observables, is zero or not. While the optimal classical post-processing for the SPT order parameter, QCNN, and Exact QCNN after measurements is determined based on Thm. 2, the same does not hold for O_{FM}. This is because, unlike other cases where the measurement results follow a Bernoulli distribution, the measurement outcomes associated with O_{FM} follow a multinomial distribution. For multinomial distributions, there is no general result providing a UMP test analogous to Lemma 2. Therefore, since knowing the order parameter implies possessing prior knowledge about quantum phases, we employ a Bayesian test to make effective use of this knowledge.

The eigenvalue decomposition of the order parameter O_{FM} is given by

$$O_{\text{FM}} = \frac{1}{L} \sum_{i=1}^{L} Z_i = \sum_{m=0}^{L} \lambda_m \Pi_m, \tag{29}$$

where $\lambda_m = 1 - 2\frac{m}{L}$ are the eigenvalues, and $\Pi_m = \sum_{|\lambda|=m} |\lambda\rangle\langle\lambda|$ represents the projection onto the subspace spanned by computational basis states with Hamming weight $|\lambda| = m$. Performing the projective measurement described by O_{FM} n times, we denote X_m as the number of times Π_m is observed and define the random variable $\mathbf{X} = (X_0, ..., X_L)$. To formalize this setting, we introduce the multinomial and Dirichlet distributions. The multinomial distribution $\text{Mul}(n, \mathbf{p})$ describes the probability of obtaining category counts $\mathbf{X} = (X_0, ..., X_L)$ given event probabilities $\mathbf{p} = (p_0, ..., p_L)$, where $p_m \geq 0$ and $\sum_{m=0}^{L} p_m = 1$. The probability mass function is

$$p_{\text{Mul}}(\mathbf{x} \mid n, \mathbf{p}) = \frac{n!}{x_0! \dots x_L!} \prod_{m=0}^{L} p_m^{x_m}, \tag{30}$$

where $\sum_{m=0}^{L} x_m = n$. The Dirichlet distribution $\text{Dir}(\alpha)$ is defined over the L-dimensional simplex $\mathbf{P} = (P_0, ..., P_L)$, where $P_m \geq 0$ and $\sum_{m=0}^{L} P_m = 1$. It is parameterized by pseudo-counts $\alpha = (\alpha_0, ..., \alpha_L)$, with the probability density function

$$p_{\text{Dir}}(\mathbf{p} \mid \alpha) = \frac{1}{B(\alpha)} \prod_{m=0}^{L} p_m^{\alpha_m - 1}, \tag{31}$$

where $B(\alpha)$ is the multivariate Beta function given by

$$B(\alpha) = \frac{\prod_{m=0}^{L} \Gamma(\alpha_m)}{\Gamma\left(\sum_{m=0}^{L} \alpha_m\right)}, \tag{32}$$

with $\Gamma(\cdot)$ denoting the Gamma function.

The hypothesis corresponding to the trivial phase (labeled $y^{(i)} = 0$) is $H_0 : \mathrm{Tr}(\rho_{\mathrm{test}}^{(i)} O_{\mathrm{FM}}) = 0$, and the true distribution of $\mathbf{X}$ follows $\mathrm{Mul}(n, \mathbf{p} = (\mathrm{Tr}(\rho_{\mathrm{test}}^{(i)} \Pi_0), ..., \mathrm{Tr}(\rho_{\mathrm{test}}^{(i)} \Pi_L)), H_0) = \mathrm{Mul}_0^{(i)}$. As a prior for the multinomial probabilities $\mathbf{P}$, we choose a Dirichlet distribution $\mathrm{Dir}(\alpha = \alpha_0, H_0)$, where

$$\alpha_0 = \frac{1}{2^L} \left(\binom{L}{0}, ..., \binom{L}{L} \right). \tag{33}$$

This choice is motivated by the fact that the trivial phase in the one-dimensional cluster-Ising model is dominated by $\sum_{i=1}^{L} X_i$, whose ground states are uniform superpositions of computational basis states. Since each Π_m represents a sum of projections onto $\binom{L}{m}$ individual computational basis states, we choose the Dirichlet prior so that α_m is proportional to $\binom{L}{m}$. The marginal likelihood under H_0 is then computed as

$$\begin{aligned} p(\mathbf{x} \mid n, H_0) &= \int p_{\mathrm{Mul}}(\mathbf{x} \mid n, \mathbf{p}) p_{\mathrm{Dir}}(\mathbf{p} \mid \alpha = \alpha_0, H_0) d\mathbf{p} \\ &= \int \frac{n!}{x_0! \dots x_L!} \prod_{m=0}^{L} p_m^{x_m} \frac{1}{B(\alpha_0)} \prod_{m=0}^{L} p_m^{\alpha_m - 1} d\mathbf{p} \\ &= \frac{n!}{x_0! \dots x_L!} \frac{1}{B(\alpha_0)} \int \prod_{m=0}^{L} p_m^{\alpha_m + x_m - 1} d\mathbf{p} \\ &= \frac{n!}{x_0! \dots x_L!} \frac{B(\alpha_0 + \mathbf{x})}{B(\alpha_0)}. \end{aligned} \tag{34}$$

On the other hand, the hypothesis corresponding to the FM phase (labeled $y^{(i)} = 1$) is $H_1 : \mathrm{Tr}(\rho_{\mathrm{test}}^{(i)} O_{\mathrm{FM}}) \neq 0$, and the true distribution of $\mathbf{X}$ follows $\mathrm{Mul}(n, \mathbf{p} = (\mathrm{Tr}(\rho_{\mathrm{test}}^{(i)} \Pi_0), ..., \mathrm{Tr}(\rho_{\mathrm{test}}^{(i)} \Pi_L)), H_1) = \mathrm{Mul}_1^{(i)}$. As a prior, we choose $\mathrm{Dir}(\alpha = \alpha_1, H_1)$, where

$$\alpha_1 = \frac{2}{(L+1)(L+2)} (1, ..., L+1) \quad \text{or} \quad \frac{2}{(L+1)(L+2)} (L+1, ..., 1). \tag{35}$$

This choice is motivated by the fact that the FM phase in the one-dimensional cluster-Ising model is dominated by $-\sum_{i=1}^{L} Z_i Z_{i+1}$, whose ground states are superpositions of $|0\rangle^{\otimes L}$ and $|1\rangle^{\otimes L}$. The observed Π_m values are thus more likely to be biased toward either small or large m. The marginal likelihood under H_1 is similarly given by

$$p(\mathbf{x} \mid n, H_1) = \frac{n!}{x_0! \dots x_L!} \frac{B(\alpha_1 + \mathbf{x})}{B(\alpha_1)}. \tag{36}$$

In Bayesian hypothesis testing, the Bayes factor is defined as the ratio of marginal likelihoods,

$$\mathrm{BF}_{10} = \frac{p(\mathbf{x} \mid n, H_1)}{p(\mathbf{x} \mid n, H_0)} = \frac{B(\alpha_1 + \mathbf{x}) B(\alpha_0)}{B(\alpha_0 + \mathbf{x}) B(\alpha_1)}, \tag{37}$$

and the test is performed by comparing BF_{10} to a threshold c. Since the prior distribution under H_1 is not uniquely determined, we adopt the Bayes factor corresponding to the distribution that deviates most from the threshold. The Type-I and Type-II error probabilities are then given by

$$\alpha_n^{(i)} = \Pr\left(\mathrm{BF}_{10} \geq c \mid \mathbf{X} \sim \mathrm{Mul}_0^{(i)}, H_0\right), \quad \beta_n^{(i)} = \Pr\left(\mathrm{BF}_{10} < c \mid \mathbf{X} \sim \mathrm{Mul}_1^{(i)}, H_1\right). \tag{38}$$

The error probabilities α_n and β_n computed in the main text are the averages of $\alpha_n^{(i)}$ and $\beta_n^{(i)}$ over the test data for each phase, respectively.

D.3 QCNN and Exact QCNN

Exact QCNN for the FM phase Exact QCNNs are constructed based on the Multiscale Entanglement Renormalization Ansatz (MERA)[13] and the Multiscale String Operator (MSO)[1,14]. The MSO is defined as a sum of products of exponentially many string order parameters that act on different locations. For example, for the SPT phase, the MSO is expressed as

$$\mathrm{MSO} = \sum_{ab} C^{(1)}_{ab} \mathscr{S}_{ab} + \sum_{a_1 b_1 a_2 b_2} C^{(2)}_{a_1 b_1 a_2 b_2} \mathscr{S}_{a_1 b_1} \mathscr{S}_{a_2 b_2} + \cdots, \tag{39}$$

where the string order parameter O_{SPT} in the main text is redefined as $\mathscr{S}_{ab} = Z_a X_{a+1} X_{a+3} \ldots X_{b-3} X_{b-1} Z_b$. The observable in the Heisenberg picture for the Exact QCNN proposed in Ref.[1] (Fig. 1(b)) matches this expression.

We consider an Exact QCNN for the FM phase characterized by the order parameter that is the linear combination of local observables, as O_{FM} in the main text. Since this order parameter is not a string, we instead consider the long-range order parameter $\mathscr{S}_{ab} = Z_a Z_b$ and construct a circuit such that the observable in the Heisenberg picture matches Eq. (39). The Exact QCNN circuit for the FM phase is shown in Fig. 6(a). The unitary operation combining the convolution and pooling layers at a certain depth d, $U^{(d)}_{\mathrm{CP}}$, is defined as shown in Fig. 6(b). Using the relations for Pauli propagation illustrated in Fig. 6(c), the observable in the Heisenberg picture for this Exact QCNN becomes

$$\begin{aligned}
&(U^{(depth)}_{\mathrm{CP}} \cdots U^{(1)}_{\mathrm{CP}})^\dagger (I \otimes \cdots \otimes I \otimes Z \otimes I \otimes \cdots \otimes I \otimes Z)(U^{(depth)}_{\mathrm{CP}} \cdots U^{(1)}_{\mathrm{CP}}) \\
=&\sum_{ab} C^{(1)}_{ab} Z_a Z_b + \sum_{a_1 b_1 a_2 b_2} C^{(2)}_{a_1 b_1 a_2 b_2} Z_{a_1} Z_{b_1} Z_{a_2} Z_{b_2} + \cdots \\
=&\sum_{ab} C^{(1)}_{ab} \mathscr{S}_{ab} + \sum_{a_1 b_1 a_2 b_2} C^{(2)}_{a_1 b_1 a_2 b_2} \mathscr{S}_{a_1 b_1} \mathscr{S}_{a_2 b_2} + \cdots,
\end{aligned} \tag{40}$$

which matches the MSO in Eq. (39).

In the main text (the subsection on error probabilities), it is evident that under a few copies of test data, the Exact QCNN for the FM phase performs worse than the order parameter. This suggests that Exact QCNNs may not be advantageous for quantum phases characterized by order parameters expressible as linear combinations of local observables.

Classical Neyman-Pearson test using QCNNs and Exact QCNNs The QCNN and the Exact QCNN classify quantum phases based on the expectation values of Pauli operators or Pauli strings with respect to the output quantum states (e.g., ZXZ Pauli string: the Exact QCNN for the Trivial vs. SPT case). By performing the projective measurement described by a Pauli operator or Pauli string n times, we test whether the output expectation value $f(\rho^{(i)}_{\mathrm{test}})$ for input test data $\rho^{(i)}_{\mathrm{test}}$ exceeds 0.5 or not. The hypothesis corresponding to the trivial phase (labeled $y^{(i)} = 0$) is $H_0 : f(\rho^{(i)}_{\mathrm{test}}) \leq 0.5$, and the hypothesis corresponding to the non-trivial phase (labeled $y^{(i)} = 1$) is $H_1 : f(\rho^{(i)}_{\mathrm{test}}) > 0.5$. The test described in case (ii) of Thm. 2 with $\langle O \rangle_{\mathrm{th}} = 0.5$ is therefore UMP for these hypotheses. We refer to this test as the classical Neyman-Pearson test for QCNNs and Exact QCNNs.

The Type-I error probability for test data labeled $y^{(i)} = 0$ is given by

$$\alpha^{(i)}_n = \sum_{x>c} \binom{n}{x} \left(\frac{1+f(\rho^{(i)}_{\mathrm{test}})}{2} \right)^x \left(\frac{1-f(\rho^{(i)}_{\mathrm{test}})}{2} \right)^{n-x} + \gamma \binom{n}{c} \left(\frac{1+f(\rho^{(i)}_{\mathrm{test}})}{2} \right)^c \left(\frac{1-f(\rho^{(i)}_{\mathrm{test}})}{2} \right)^{n-c}, \tag{41}$$

and the Type-II error probability for test data labeled $y^{(i)} = 1$ is given by

$$\beta^{(i)}_n = \sum_{x<c} \binom{n}{x} \left(\frac{1+f(\rho^{(i)}_{\mathrm{test}})}{2} \right)^x \left(\frac{1-f(\rho^{(i)}_{\mathrm{test}})}{2} \right)^{n-x} + (1-\gamma) \binom{n}{c} \left(\frac{1+f(\rho^{(i)}_{\mathrm{test}})}{2} \right)^c \left(\frac{1-f(\rho^{(i)}_{\mathrm{test}})}{2} \right)^{n-c}. \tag{42}$$

The error probabilities α_n and β_n computed in the numerical results are the averages of $\alpha^{(i)}_n$ and $\beta^{(i)}_n$ over the test data for each phase, respectively.

References

1. Cong, I., Choi, S. & Lukin, M. D. Quantum convolutional neural networks. Nat. Phys. **15**, 1273–1278 (2019).
2. Hastings, M. B. & Koma, T. Spectral gap and exponential decay of correlations. Commun. Math. Phys. **265**, 781–804 (2006).
3. Nachtergaele, B. & Sims, R. Lieb-robinson bounds and the exponential clustering theorem. Commun. Math. Phys. **265**, 119–130 (2006).

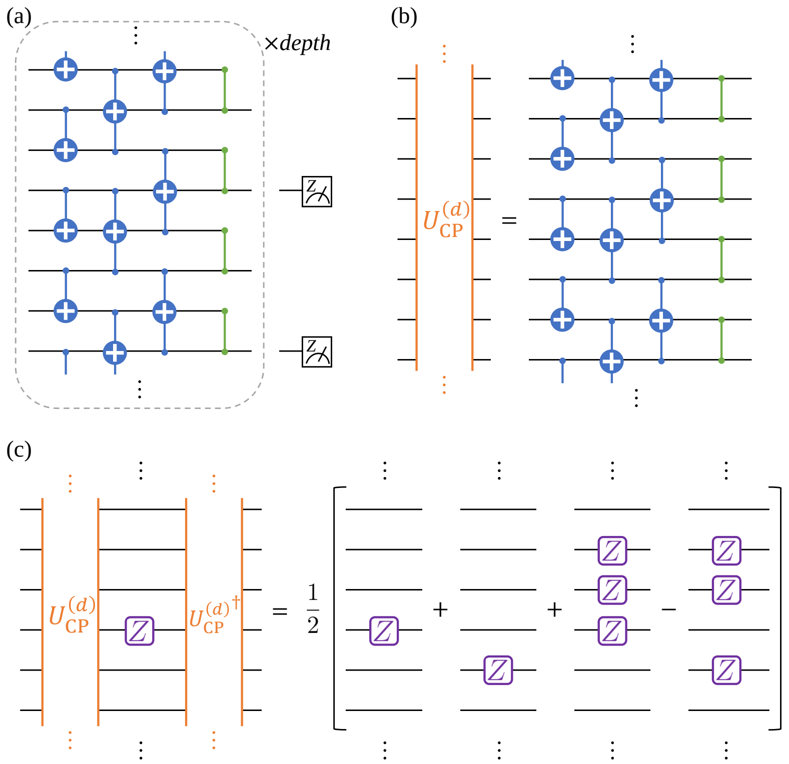

Figure 6. Exact QCNN for the FM phase. Panel (a) shows the full Exact QCNN circuit, identical to Fig. 1(a), where the convolution and pooling layers at a certain depth d are denoted as $U^{(d)}_{\mathrm{CP}}$, as shown in panel (b). Panel (c) illustrates the equality $U^{(d)\dagger}_{\mathrm{CP}} Z_i U^{(d)}_{\mathrm{CP}} = \frac{1}{2}(Z_i + Z_{i+1} + Z_{i-2}Z_{i-1}Z_i - Z_{i-2}Z_{i-1}Z_{i+1})$ obtained using Pauli propagation. The blue two-qubit gates represent controlled–X gates, the blue three-qubit gates represent Toffoli gates, the green two-qubit gates represent controlled–Z gates, and the ZZ measurement indicates a projective measurement in the computational basis.

4. Beck, M. & Robins, S. Computing the Continuous Discretely (Springer New York, 2015), 2 edn.

5. Liggett, T. M. Ultra logconcave sequences and negative dependence. J. Comb. Theory. Ser. A **79**, 315–325 (1997).

6. Johnson, O., Kontoyiannis, I. & Madiman, M. Log-concavity, ultra-log-concavity, and a maximum entropy property of discrete compound poisson measures. Discret. Appl. Math. **161**, 1232–1250 (2013).

7. Sander, L. C., McMahon, N. A., Zapletal, P. & Hartmann, M. J. Quantum convolutional neural network for phase recognition in two dimensions (2024). Preprint at http://arxiv.org/abs/2407.04114.

8. Dusuel, S., Kamfor, M., Orús, R., Schmidt, K. P. & Vidal, J. Robustness of a perturbed topological phase. Phys. Rev. Lett. **106**, 107203 (2011).

9. Fisher, R. A. The design of experiments (Oliver Boyd, 1935).

10. Neyman, J. & Pearson, E. S. Ix. on the problem of the most efficient tests of statistical hypotheses. Philos. Transactions Royal Soc. London. Ser. A, Containing Pap. a Math. or Phys. Character **231**, 289–337 (1933).

11. Karlin, S. & Rubin, H. The theory of decision procedures for distributions with monotone likelihood ratio. The Annals Math. Stat. **27**, 272–299 (1956).

12. Lehmann, E. L. & Romano, J. P. Testing Statistical Hypotheses (Springer Science Business Media, 2006).

13. Vidal, G. Class of quantum many-body states that can be efficiently simulated. Phys. Rev. Lett. **101**, 110501 (2008).

14. Lake, E., Balasubramanian, S. & Choi, S. Exact quantum algorithms for quantum phase recognition: Renormalization group and error correction. PRX Quantum **6**, 10350 (2025).
